# Supplementary material for: Specimen sharing for epidemic preparedness: Building a virtual biorepository system from local governance to global partnerships
Source: PLOS Glob Public Health. 2023 Oct 11;3(10):e0001568. doi: 10.1371/journal.pgph.0001568 (PMC10566708; doi:10.1371/journal.pgph.0001568)
Supplement: S1 Table — (DOCX) [file pgph.0001568.s001.docx]

| **S1 Table. Virtual Biorepository Benefits Questionnaire** | |
| --- | --- |
| **CATEGORY** | **QUESTIONS** |
| **Profile of respondents** | - 1. What is the nature of your institution |
|  | - 1. What is your role |
|  | - 1. What is your geographic location |
| **Benefits of participation: support functions and resources** | - 1. Which planned VBR benefits would be most important to you/your institution? |
|  | - 1. Which VBR support functions would be most valuable to your current biorepository or collection? |
|  | - 1. Which additional resources would allow you to participate in the VBR? |
| **Benefit sharing and the Nagoya protocol** | - 1. Does your country have a specific policy in accord with the NP? |
|  | - - - - If yes, would it prevent you from sharing specimens outside the country? |
|  | - - - - If yes, would you be willing to test in country? |
| **Interest in participation** | - 1. Would you be interested in participating in a VBR |
| **Contributing to reference material and panels** | - 1. Would you be willing to set aside …? |
| **Other benefits?** | - 1. Comments |
