# Supplementary material for: Specimen sharing for epidemic preparedness: Building a virtual biorepository system from local governance to global partnerships
Source: PLOS Glob Public Health. 2023 Oct 11;3(10):e0001568. doi: 10.1371/journal.pgph.0001568 (PMC10566708; doi:10.1371/journal.pgph.0001568)
Supplement: S2 Table — (DOCX) [file pgph.0001568.s002.docx]

**S2 Table. Interviews to assess needs and barriers to accessing specimens**

| 1. **What are your greatest hurdles for access to specimens?**   *Examples of issues to consider*: identifying sources; reliability of sources; cost;  quality of specimens; completeness and reliability of accompanying data; legal hurdles: Material Transfer Agreements (MTA) and negotiations; other |
| --- |
| 1. **What are your current strategies for access?** |
| 1. **What type of specimens have been difficult to source for COVID/19 and other infectious diseases?** |
| 1. **What are some of the challenges for access to specimens in LMICs?** |
| - 1. **Which features of a Virtual Biorepository would be most useful?**   *VB features to consider*: coordinated, one stop access to specimens; an online catalog or directory of sources; more affordable specimen sources; trusted source of qualified specimens; facilitated access including MTA negotiations and logistics; other |
| - 1. **Would you be willing to contribute to creation of a manufacturer’s panel ^(1^** |
| **7.Do you have any additional comments and recommendations?** |
