# Supplementary material for: Specimen sharing for epidemic preparedness: Building a virtual biorepository system from local governance to global partnerships
Source: PLOS Glob Public Health. 2023 Oct 11;3(10):e0001568. doi: 10.1371/journal.pgph.0001568 (PMC10566708; doi:10.1371/journal.pgph.0001568)
Supplement: S3 Table — (DOCX) [file pgph.0001568.s003.docx]

| S3 Table Interview summary of gaps and barriers to access | | | | |
| --- | --- | --- | --- | --- |
| Grouping | Commercial Diagnostics Developers | | Not-for-profit | |
|  | **Small scale* IVD companies** | **High volume** IVD companies** | **Specimen providers and services** | **Public Health/ government collaborations** |
| Access to specimen strategies | - Commercial specimen supplier - Government/ academic collaborations | - Established collaborations, with long term agreements - Use some commercial sources (via NGOs in LMICs) | Collaborations and partnerships | Asking other laboratories/  academic networks |
| Greatest barriers to access *** | - Identifying reliable sources/timely access - Quality and quantity of specimens - Access to data and quality of data | - Quality of specimens - Access to complete accompanying data - Data access restrictions (Europe) | - Access to complete accompanying data, especially for specimens left over from testing - Pathogen characterization/   sequence data   - Legal negotiations | - Access to specimen sources - Access to clinical data (where/when specimens were collected) - Timely access |
| Availability of specific specimen types (COVID-19 and other)*** | - Timely availability early in an outbreak/epidemic - Limited availability of non-COVID 19 specimens (seasonal, geographic, etc.,) - COVID-19: swabs, virus, longitudinal samples | Access to specimen types needed for development | - Scarcity of specimen types needed at different stages of an outbreak or epidemic and diseases other than COVID-19 - Access to clinical trial specimens | - Specific types needed based on pathogen and stage of outbreak; - Peripheral blood mononuclear - Longitudinal samples |
| Gap for IVD quality assessment*** | - Panels and reference materials - Panels to meet regulatory requirements | Panels and reference materials | Panels and reference materials | Panels and reference materials |
| Access to specimens in LMICs*** | Not often, through desired. Not enough resources | Differences in country regulations (e.g., Nagoya protocol) regarding sharing and biosafety | Difficulties in both exporting and importing specimens | - Biosafety and security restrictions - Infrastructure needed to maintain quality and safety |
| VBR solutions – features of VBR to facilitate access | - Directory - Coordinate a one- stop, trusted resource - Accelerating access: logistics, negotiations, etc. - Access to rare -disease specimens | - Access to well characterized specimens - Access to complete specimen-associated data - Trusted resource to accelerate development path and approval by regulatory authority - Access to a directory of sources | Both groups in the not-for profit category identified same benefits   - Access to sufficient volumes for panels - Standardized templates for agreements - Harmonization of initiatives and standardized vocabulary - Responsive to needs of stakeholders/users of specimens - Governance: based on local ownership and equal partnerships - Building trust through quality - Benefits and capacity building in LMICs (training, other resources) - Access to well characterized and   well annotated specimens | |

*Small scale companies are ones that sell 10 or less IVD products with sales of less than 1% of IVD market

**Large volume producers with over greater than 10 IVD products and sales of over 10% of IVD market (Michell)

*** Types of barriers that delay and preclude accurate and timely test development-to-market timeline
