# Supplementary material for: Specimen sharing for epidemic preparedness: Building a virtual biorepository system from local governance to global partnerships
Source: PLOS Glob Public Health. 2023 Oct 11;3(10):e0001568. doi: 10.1371/journal.pgph.0001568 (PMC10566708; doi:10.1371/journal.pgph.0001568)
Supplement: S1 Fig — A. The Industrial University of Santander, Bucamaranga, Colombia and the Fundaciôn INFOVIDA and Centro de Atención y Diagnóstico de Enfermedades Infecciosas-CDI. B Cayetano Heredia University, Lima, Peru. C. University of Carabobo, Valencia, Venezuela. (DOCX) [file pgph.0001568.s004.docx]

S1A Fig. The Industrial University of Santander, Bucamaranga, Colombia and the Fundaciôn INFOVIDA and Centro de Atención y Diagnóstico de Enfermedades Infecciosas-CDI.

S1B Fig. Cayetano Heredia University, Lima, Peru

S1C Fig. University of Carabobo, Valencia, Venezuela
