# Supplementary material for: Specimen sharing for epidemic preparedness: Building a virtual biorepository system from local governance to global partnerships
Source: PLOS Glob Public Health. 2023 Oct 11;3(10):e0001568. doi: 10.1371/journal.pgph.0001568 (PMC10566708; doi:10.1371/journal.pgph.0001568)
Supplement: Attachment — Submitted filename: PGPH-D-22-02118_Response to Reviewers_21 May 2023.pdf [file pgph.0001568.s005.pdf]

Response to Reviewers

Manuscript PGPH-D-22-02199

21 May 2023

Reviewer 1

Dear authors,
compliments for your study. I think it is very useful for the scientific community.
To report only a few punctuation and typing errors.

Thank you for your review. We corrected the punctuation and typing errors pointed out (line 402 and 442-443 and spacing error for line 475 of the original manuscript). For lines 442-443 revised and divided into shorter sentences instead of dashes. The corrections are now found in line 375, line 431-432 in the revised manuscript.

Reviewer 2:

The authors propose a Virtual Biorepository System (VBS) as a potential solution to global specimen sharing and epidemic/pandemic responsiveness. I appreciate the goals and sentiment and their call for increased support of biorepositories particularly in LMICs, but overall, I found the perspective narrow and did not see how the VBS framework differs from existing tools and protocols. For example, lines (L) 327-332 exactly describe the formal cataloging process, public databasing, and specimen loan procedures of natural history museums, but with no reference to that community or their published SOPs/best practices. I encourage the authors to expand the perspective to the wealth of literature emanating from non-human biorepositories, see: Bakker et al. 2019, Galbreath et al. 2019, Phillips et al. 2019, Brooks et al. 2020, Colella et al. 2020, NASEM 2021, Theirs et al. 2021, Thompson et al. 2021, Adler Miserendino et al. 2022, Hoberg et al. 2022, among others. Last, it's unclear how (or if) VBS will plug into existing biorepository database infrastructure (e.g., Arctos, Specify, Symbiota), established data standards (e.g., DarwinCore), recent efforts to inventory global tissue resources (e.g. GGBN), or if VBS intends to reinvent the wheel?

It is not the intent of our manuscript to explore universal biorepository solutions, we have edited language in the manuscript to clearly say that ours is about access to specimens for outbreak-prone infectious disease and ours is meant to fill in gaps and synergize with other infectious disease specimen collections.

Thank you for your review and references, we appreciated the point of view about the need for more inclusiveness and collaboration between biodiversity/natural history and biomedical biorepositories that have both developed standards and solutions which could be mutually beneficial. We included the reference by Coletta et.al that you recommended (line 51) to acknowledge that similar solutions have been considered by biodiversity/natural history biorepository collections.

The authors reviewed a large body of work of peer reviewed publications and initiatives related to biomedical biorepositories and more specifically biorepositories for infectious disease and global public health research, cited throughout the manuscript. Our review of biorespositories in the Introduction

section is meant to give a background understanding of the extensive work and improvement of biorespository sciences.

Response to issues identified as major:

The manuscript almost exclusively addresses commercial uses of biological materials and I wish the authors were more candid about that focus in the abstract/intro, possibly even in the title. Otherwise, how will non-commercial research be accommodated? For example, framing biological materials within 'a federated system [based] on a demand-and supply model' (L476) may make sense for commercial establishments, but it positions biological samples as a commodity that is then bartered and traded by governments. Such a model unintentionally excludes non-commercial researchers and organizations (e.g., universities), particularly those from LMICs, from participation due to 'buy in' requirements. How will this model be any different?

Focus and scope*:*

It is not the intent of our manuscript to explore universal biorepository solutions, we have revised the manuscript to point this out more clearly in lines 46-51 and in lines 407-414

We responded to the comment about inconsistency between reference to biorepositories by further clarifying our focus on biorepositories for specimens from infectious diseases lines 441-445.

Commercial uses:

There is absolutely no implied intent in the manuscript to prioritize commercial uses.

For development and validation of diagnostics and vaccines, collaboration between academic researchers, government and not-for profit organizations and commercial entities are required; finding out what, if any issues IVD developers have for access to high quality specimens and reference materials, as lack of access may result in tests that does not work for the intended uses. We invited commercial entities because the research and public health sectors, do not often seek IVD developers for input thus there are areas of mistrust and misunderstanding. We believe this interaction is important to align the needs of both specimen users and providers openly.

A functioning collaborative example is provided in the case of Lyme disease serodiagnosis (reference 24).

Revised the statement **"**on demand and supply model" to clarify that instead of the most common current paradigm of large, centralized collections that are costly to maintain, the federated system proposed for the VBS will make locally held specimens available for sharing when needed. We included a reference to Coletta to show that such models have been considered for biodiversity biorepositories.

The desired outcome of the VBS is to build capacity in LMICs for biorepositories and laboratories needed for research and diagnostics adhering to International ethical standards and local ethical oversight of human specimens to be incorporated into the VBS governance, would eliminate the concern about misuse (bartering) of specimens. Please see also below in context of the Zika Alliance governance models.

Focus on serology:

As mentioned above, the intent is to solve a specific problem, and complement other initiatives. There are other initiatives focused on pathogens mentioned in the manuscript, such as the EVAg and WHOs BioHub. It is not our intent to limit to serology, but it was both an identified unmet need for evaluation of diagnostics and vaccines and a practical first approach to operationalizing and testing the concept.

Zika Alliance governance

The design of the VBS is a departure from centralized biorepositories more typical of "biomedical" human specimen biorepositories, with centralized maintenance of specimens, oversight, and decision processes. We interviewed the existing ZikAlliance biorepository members with intent only to understand their governance and function. We asked how their system worked and could consider the use of their model In the proposed VBS disseminated model the stewardship (or ownership) of specimens remains local in compliance with institutional and national requirements. The governance models included in the supporting material are meant to illustrate the local ownership regarding ethical oversight and decision process for sharing; the detailed examples in Supplements illustrate commonalities and differences between the three institutions and countries mentioned in the body of the manuscript.

Survey methods

The survey was disseminated by email and posting on the The Global Health Network website. We don't have the information or a way to answer the selection of "other". When "other" was selected, an opportunity for text description was provided.

Issues identified as Minor

Revised throughout by final grammar and spelling check in WORD

Substituted: "specimen associated data" for "annotated"

Line 23: What kind of barriers.

Revised. Barriers were identified in the questionnaire and interview results.

L32*: Add Philips et al. 2019 as an example of best practices, particularly for cryogenic tissue collections. American Society of Mammalogist's Systematic Collection Committee is another accrediting institution.*

Ours is focused on human specimens, therefore it is appropriate to use ISO as an accepted comprehensive accreditation system for biomedical repositories and laboratories; the College of American Pathologists would be another for human specimens, based on ISBER best practices cited in Reference 11.

L34-36: *Has this been quantified? There are also very large natural history biorepositories (100s of thousands to millions of samples/specimens) developed for other purposes that are also available for disease research.*

Changed to many, instead of most; supported by references cited added Mendy et.al. as a more recent reference, this is in line 73 in the revised manuscript

L62-63: All biological materials are subject to the Nagoya Protocol, not just those that contribute Â'to the development of products'.
Inserted "including" but not exclusive…line 64 in the revised version of the manuscript

L101: "for the "global good" has been used by commercial enterprise rationalize resource exploitation of LMICs and is why the Nagoya Protocol exists. Please rephrase.
Thank you for pointing this out. We have chosen to rephrase this as a "global public good" using the International Monetary Fund definition to be more precise. The new phrase indicates here that the benefits ought to affect all citizens of the world that encompasses many aspects of our lives, including health. Changes are made then in the title page, Abstract (line 2), Introduction (line 104), in the Conclusion (line 465) in the revised manuscript.

L404-418 echo the goals of the Project ECHO Museums and Emerging Pathogens in the Americas (MEPA) Community of Practice. Consider citing Colella et al. 2021:
Included Collela at reference; Reference 11, generic to Best Practices ; more appropriate for our scope are clinical laboratory standards

L418: remove 'specifically;
Revised and removed

Line 466: Are all clients commercial
No, all users are included. There are no "clients" here only those who are specimen users and contributors , that was our mistake and thank you for point that out, we have removed "clients" and instead used "specimen users" to include all parties. We chose to engage with commercial entities only because of the nature of the VBS, which is a de-centralized approach where each local site maintains stewardship of the specimens then have and can choose selected specimens to share. Often the view of an IVD-commercial, for profit/non-profit; academic; governmental entities are not sought together. We included the commercial entities to learn of how they approached acquiring specimens and what the challenges have been. In the end, the goal is to shorten the time to validating assays, evaluate vaccines/therapeutics and conduct research. During COVID-19, the partnership of all entities was evident.

Figure 1 does not add the main message of the manuscript – all values are reported in text.
Added as a visual summary, for emphasis; text added additional information

SI Figure 1A-C text need to be enlarged. What do the colors mean?
The colors are to denote the different entities involved. The authors wish to keep them as it is for their guidance reference.

All there tables seem unnecessary
Included as a summary, for clarity and emphasis

On line 48 on the Manuscript, LIMC Acronyms was used for the first time without writing it in full.

Has been corrected

Reviewer 3:

The manuscript is a qualitative research article developed from the analysis of products of interviews, workshops, and survey/questionnaires on proposed Virtual Biorepository System (VBS) framework for specimen sharing for outbreaks preparedness. Generally, the sample size ie number of individuals, institutions, organizations and countries' opinion sampled were low to support effectively the authors' objective of establishing the VBS framework for specimen sharing.
The objective is noble and seriously needed to strengthen the global fight against future outbreaks, as such this work can serve as foundation for further research on VBS, which the researchers would recruit, collect and analyze larger and more complex data from more individuals, institutions, organizations and countries to develop the VBS framework.
The following were some other review comments for the authors attention:

We thank the reviewer for supporting our goal and concur that manuscript is an initial qualitative study, to investigate interest and acceptance of the concept of the proposed virtual biorepository system, to be followed by further studies to refine and operationalize the proposed approach.

In line 48 of the manuscript. LIMC acronym was used for the first time without writing it in full.
Addressed, corrected at Line 48 and corrected throughout the manuscript using “FIND” function.

On line 68- 70, a categorical statement was made without any reference being cited.
A reference was added by Mendy et.al, in the revised manuscript to be found on Line 73

On line 92, ASTHM acronyms was also used for the first time without writing it out full:
Was corrected at Line 94 in the revised manuscript

On line 149, the methodology of the study left out questions around opinion of respondents on VBS as it affects their countries' national biosecurity.
The intent was to ask about “specific hurdles” they interviewees may have encountered in sourcing specimens, we did not target question regarding whether sharing specimens would affect their national biosecurity, this is found in Line 150 in the revised manuscript. We will address national biosecurity issues in our follow up when we can define more clearly the role biosecurity may play in sharing specimens.

On line 152 - 153, Categories of diagnostics industry companies were used in the paper without citing any reference.
Please see Reference 22. Now found in Line 141 in the revised manuscript.

On line 154, the geographical coverage of the entities interviewed was not clear. Was their coverage global or restricted. This could skew findings/opinion and render it not representative enough.
Added a sentence regarding geographic location and reach of companies interviewed. This can be found in Line 145 in the revised manuscript.

Understandably companies agreed to participate if not identified by name: of the companies mentioned two have international reach and international subsidiaries and one of these is included in the 10 largest diagnostics companies; one of the small companies provides quality evaluation tools; and the other three companies either provide specialized test platforms or have a limited numbers of products. Included are US, Europe and Canada based in vitro diagnostic providers.

On line 178, Don't you think this number of respondents (47) is too low to power the deductions from the survey, especially as they also have wide characteristic base.
We agree, it is an initial qualitative study, and we wished to have had more respondents. We launched at the time when COVID-19 Public Health Emergency of International Concern was invoked, we communicated right away to ask participants to reply and reminded them 4 times (Please see the Limitations section). The purpose was to gather information: assess interest and acceptance of the VBS concept, complementing the information obtained in workshops mentioned and to inform next steps.

Throughout this paper, the authors chose to use a mix of the word Specimen and Sample in the manuscript Â– will it not be better to stick to one nomenclature, unless each word carries different meaning where it was used which may be difficult to proof in this paper.
Corrected: specimens is used throughout

On line 265, Market size - Can you insert reference for the classification of the in vitro diagnostics into the different market sizes used in this manuscript
Reference 22 is widely used and cited but it only lists the largest IVD companies, we moved the word "market" to avoid confusion, simply to say they were of differing sizes in line 247 in the revised manuscript.

On line 301, correct the "European counties" in the manuscript to "European countries".
Was Corrected, it is found in line 279 in the revised manuscript

On line 320, Consortium was cancelled - Was it not currently in place or Is it a suggestion for future implementation.
Corrected- deleted consortium.

On line 332, The following statement is missing some words, as such it's not reading well - "the PI the forwards it for to the next level to the regulator".
Corrected; We changed this to "Approved requests were forwarded to the regulatory agency,…." This can be found in Line 309 in the revised manuscript.
